# Supplementary material for: Are carbon emissions trading and green financial instruments synergistic? -Comprehensive quantitative research based on content analysis
Source: PLoS One. 2024 Mar 7;19(3):e0298601. doi: 10.1371/journal.pone.0298601 (PMC10919601; doi:10.1371/journal.pone.0298601)
Supplement: S1 Data — (ZIP) [file pone.0298601.s001.zip › Data/地级市Z_F工作报告.zip!/╜¡╦╒/╦╒╓▌╩╨/└┤╫╘╣1⁄2╓┌║┼í╛┬φ┐╦╩2╛▌═°í┐_2014.docx]

各位代表：

　　现在，我代表苏州市人民政府，向大会报告工作，请予审议，并请市政协各位委员和其他列席人员提出意见。

　　一、2013年工作回顾

　　过去的一年，面对复杂严峻的经济形势，面对日趋激烈的竞争环境，面对艰巨繁重的工作任务，市政府在中共苏州市委的正确领导下，认真学习贯彻中共十八大和十八届三中全会精神，围绕市十五届人大二次会议确定的目标任务，紧扣科学发展主题和转变经济发展方式主线，突出创新引领、聚焦转型、生态优先、民生为本等重点，团结依靠全市人民，真抓实干，克难奋进，推动“三区三城”建设和率先基本实现现代化取得了新的进展。

　　1.经济运行稳中有进。全市完成地区生产总值1.3万亿元（预计数，下同），比上年增长9.8%；地方公共财政预算收入1331亿元，增长10.5%；全社会固定资产投资6002亿元，增长14%。工业经济平稳增长，总产值达到3.6万亿元，增长3.4%；实现制造业领域新兴产业产值1.4万亿元、高新技术产业产值1.3万亿元，占规模以上工业的比重分别提高到45.2%和43%。实施万企升级行动计划，完成技术改造投资1580亿元，占工业投资的比重达到65%。苏州高新区成为国家新型工业化产业示范基地，新增省级信息化与工业化融合示范区1个、示范试点企业114家。注重培育骨干企业，新增地标型企业4家。服务业提档升级，增加值增长10.7%，占地区生产总值的比重达到45.7%。苏州工业园区、昆山花桥国际商务城获批国家现代服务业综合试点。金融业规模持续扩大，新增全社会融资4000亿元，金融总资产达到3万亿元，增长12%。商贸业稳步发展，完成社会消费品零售总额3630亿元，增长13%。旅游业优化发展，实现总收入1569亿元，增长14%。吴中太湖旅游区、常熟沙家浜虞山尚湖旅游区获评国家5A级景区。国家城市旅游融合发展示范区创建工作积极开展。文化产业做大做强，主营业务收入超过3000亿元，增长19.2%。软件产业产值达到1262亿元，增长25.1%。农业现代化大力推进，新增现代农业园区面积7千公顷、高效设施农业面积4.1千公顷，高标准农田比重、农业综合机械化水平分别达到65%和87%。张家港市常阴沙现代农业产业园被认定为国家农业产业化示范基地。加快构建现代农业经营体系，专业大户和合作经济组织成为重要经营主体。地方粮食储备总量继续增加，新建域外粮食生产基地6.7千公顷。

　　2.开放创新取得成效。推动开放型经济转型升级，不断提高经济国际化水平。对外贸易保持增长，完成进出口总额3093亿美元。实现服务外包接包合同额85.8亿美元、离岸执行额46.1亿美元，分别增长53.2%和51.1%。苏州被列为国家跨境贸易电子商务服务试点。提升择商选资质量，服务业实际利用外资占到39.3%，新兴产业和高技术项目实际利用外资占到38.9%。第十五次中新联合协调理事会顺利举行，合作领域向现代服务业、科技创新和社会管理拓展。昆山深化两岸产业合作试验区和太仓港综合保税区获国务院批准设立，浒墅关经济开发区升格为国家经济技术开发区，张家港汽车整车进口口岸封关运作。中方境外协议投资额达到16.2亿美元，增长32.5%，总量实现全省“十连冠”。南北挂钩合作积极开展，苏州宿迁工业园区在全省共建园区考核中保持第一。对口扶持贵州铜仁、援建西藏林周、合作共建新疆霍尔果斯经济开发区等工作扎实推进。国际友好交往更加频繁，成功承办第四届中欧政党高层论坛和落实中国—东盟《南海各方行为宣言》第六次高官会、亚洲相互协作与信任措施会议高官会。对台合作交流取得新成果，我市成为赴台个人游试点城市。新增2个全国社区侨务工作示范单位，昆山经济技术开发区成为海外人才中国创业示范基地。

　　持续深化各项改革，不断创新体制机制。事业单位分类改革稳步推进。医疗卫生体制改革步伐加快，常熟市、太仓市县级公立医院综合改革试点认真开展。国有经济优化提升，全市国有资产总量突破1万亿元。营业税改征增值税试点工作持续深化，共计减轻纳税人负担33.5亿元。金融创新成效明显，我市获准开展商业保理试点和跨国公司总部外汇资金集中运营管理试点，全国股转系统首个路演分中心落户苏州高新区；新增金融机构38家、上市公司3家。民营经济健康发展，私营企业和个体工商户注册资本由年初的8716亿元增加到9954亿元。

　　大力推动科技创新，加快建设国家创新型城市。加大研发投入力度，全社会研究与试验发展经费支出占地区生产总值的比重达到2.6%。大中型内资工业企业研发机构基本实现全覆盖。国家重大科技基础设施纳米真空互联实验站和中国医学科学院系统医学研究所启动建设。产学研合作不断深化，苏州纳米科技协同创新中心列入国家首批高等学校创新能力提升计划。苏州自主创新广场建成启用。科技与金融结合更加紧密，“科贷通”共计为中小科技企业发放贷款80.9亿元；苏州高新区成为全国首个保险与科技结合综合创新试点单位。我市连续七次获得全国科技进步先进市称号。实施以质取胜、品牌发展和技术标准战略，创建全国质量强市示范城市进展顺利，国家城市综合服务标准化试点一期项目基本完成。专利申请量、授权量均保持全国城市第一位，苏州市和昆山市成为全国版权示范城市，常熟市和昆山市成为国家知识产权示范城市，太仓新区国家先进制造技术国际创新园获得科技部认定。统筹抓好各类人才队伍建设，新增国家“千人计划”人才36人，累计达到141人，其中创业类人才96人，位居全国城市首位；新增省“双创计划”人才102人，累计达到403人，连续七年保持全省第一。国际精英创业周系列活动取得新成效，首届技能英才周顺利举办，全国地级市中首个一园多区的国家级人力资源服务产业园落户苏州。

　　3.城乡一体步伐加快。新一轮苏州市城市总体规划研究工作有序开展，苏州历史文化名城保护规划和高铁新城总体规划编制完成。东部综合商务城、西部生态科技城、南部太湖新城、北部高铁新城建设进程加快，姑苏区资源优化整合初见成效，吴江区对接融入主城明显加速，“苏州湾”成为城市建设新亮点。苏州火车站综合改造全面竣工。太仓港疏港高速公路建成通车，张家港疏港高速公路、常嘉高速公路昆山至吴江段、312国道苏州段分流线加紧建设。中环快速路及东环、南环、西环快速路延伸线工程抓紧实施。相城大道扩建基本完工，一批城市道路综合整治工程顺利完成。轨道交通2号线投入试运营，4号线及支线、2号线延伸线和苏州高新区有轨电车1号线建设积极推进，上海轨道交通11号线昆山花桥延伸线建成运营。苏南运河苏州段和杨林塘、申张线航道整治完成年度任务。《苏州港总体规划（2013～2030）》获得交通运输部、省政府联合批复。苏州港新增万吨级以上泊位6个，完成货物吞吐量4.5亿吨，增长9.3%。特高压直流输电工程运行效益显著，智能电网加快建设。苏州工业园区、昆山市和吴中太湖新城被列为国家智慧城市试点。新增人防设施100万平方米。垃圾焚烧发电三期和餐厨垃圾收集处置二期工程投入使用。城市环境整治提升和环境卫生全民大行动取得实效，交通秩序、运管服务、人居环境、市容市貌得到改善。健全数字城管体系，网格化、精细化管理不断强化。

　　完善政策制度框架，城乡一体化发展水平进一步提高。争创国家城乡发展一体化综合改革试点工作取得成果。城乡布局和形态继续优化，土地规模经营比重、工业企业集中度、农民集中居住率分别达到91%、92%和52.2%。农村集体经济发展壮大，总资产突破1350亿元，村均收入超过650万元，分别增长12.5%和11.7%。新型合作经济组织达到4168家，持股农户比例超过96%，收益分配机制逐步完善。公共财政对“三农”的投入持续增加，强农惠农政策有效落实，农业保险承担风险保障32.3亿元，农业担保金额达到262.9亿元。农村水利建设高标准推进，轮浚河道1788条，加固圩堤137公里。村庄环境整治任务全面完成，并达到省级标准。积极开展美丽镇村建设，示范点环境面貌明显改观，吴江区同里镇入选全国首批美丽宜居小镇。

　　4.生态环境持续改善。切实保障饮用水安全，集中式饮用水水源地水质达标率保持100%。全市同城第二水源地建设和自来水厂深度处理工艺改造基本完成。东太湖综合整治、阳澄湖生态优化实现年度目标。整治农村黑臭河道201条，古城区河道“自流活水”工程投入运行。新建和改建污水处理厂21座，新增污水日处理能力52万吨。建立环境空气质量监测与信息发布系统，实施大气污染防治工程110项。治理噪声污染，声环境功能区噪声达标率保持100%。推行生活垃圾分类收集，新增试点237个。加大节能减排力度，单位地区生产总值能源消耗和主要污染物排放继续下降。注重发展循环经济，我市被评为国家循环经济示范城市，张家港市再制造产业基地成为首批国家示范基地，新增国家再制造试点企业2家。积极盘活存量用地，优化配置建设用地，土地节约集约利用水平进一步提高，吴中区成为全国国土资源节约集约模范区。认真实施生态文明建设规划，全面启动生态文明建设“十大工程”。优质水稻、特色水产、高效园艺、生态林地“四个百万亩”全部落地上图。划定了生态红线区域。完善生态补偿政策，补偿标准总体提高50%。环古城河、大运河、胥江环境综合整治取得阶段性成果，灵岩山、天平山及吴中区木渎镇周边地区环境整治生态提升工程有力推进。上方山石湖生态园、虎丘湿地公园等项目建设步伐加快，常熟市沙家浜湿地公园、吴中区太湖三山岛湿地公园升格为国家湿地公园。市区新增绿地505万平方米，农村新增林地绿地4.4千公顷，全市陆地森林覆盖率达到28.67%。我市建成国家生态城市群，并被确定为国家水生态文明试点市。

　　5.社会建设不断加强。优化配置教育资源，我市成为全国首批义务教育发展基本均衡地级市。新建和改扩建幼儿园101所、中小学64所，市属职业教育实训基地如期建成，苏州高等幼儿师范学校迁建工程开工建设。昆山杜克大学、苏州—牛津大学研究中心、新加坡国立大学苏州研究院和东南大学—蒙纳士大学苏州联合研究生院相继成立。落实教育信息化五年行动计划，信息技术应用水平得到提升。优化文化设施布局，提高文化服务质量，我市成为国家公共文化服务体系示范区。虎丘地区综合改造和桃花坞历史文化片区、天赐庄片区、渔家村越城遗址、同里历史文化遗产保护整治等工程全面推进，古城墙保护修缮二期工程如期竣工，古建老宅保护修缮取得进展。成功主办第十三届中国戏剧节。“忆江南”苏州园在第九届中国（北京）国际园林博览会上获得展园综合大奖等多个奖项。大运河苏州段申报世界文化遗产通过国际古迹遗址理事会考察评估。新一轮《苏州市志》基本完成编纂。城乡社区卫生服务普及率达到100%，社区卫生信息系统实现优化升级。市立医院本部门诊急诊楼、市社区卫生临床检验集中检测中心和苏州高新区明基医院建成启用，苏州大学附属儿童医院园区总院主体工程基本完工，市公共医疗中心开工建设。我市被赋予国家药品进口口岸功能。人口基础信息库一期工程基本建成。计划生育家庭特别扶助对象帮扶力度加大，人口和家庭公共服务进一步拓展。落实体育惠民措施，全民健身活动蓬勃开展。在第十二届全国运动会上，我市运动员获得金牌数名列全省第一。成功承办环太湖国际公路自行车赛等重大赛事。市网球中心、儿童业余体校全面建成。民族宗教工作取得新成绩。妇女儿童合法权益得到维护。支持驻苏部队建设，落实优抚安置政策，新型军政军民关系更加巩固。

　　强化文明城市长效管理，新增一批省级文明单位、文明村镇、文明社区，我市在全国城市文明程度指数测评中继续排名靠前。提炼确定的“苏州精神”更加深入人心。科普提升行动取得成效，公民科学素质指标位居全国前列。哲学社会科学研究再创优秀成果。抓好基层法治创建工作，张家港市和吴江区被评为创建全国法治县（市、区）先进单位。“六五”普法深入开展，法治文化建设走在全国前列。太仓市“政社互动”经验在全省推广。依法组织第五届社区居委会和第十届村委会换届选举。加强“平安苏州”建设，我市获得全国社会管理综合治理优秀城市“六连冠”，第三次荣获“长安杯”，太仓市成为全省首个捧得“长安杯”的县（市）。深化“平安中国”建设工作会议在我市召开，苏州经验得到推广。完善应急处置机制，科学应对和妥善处理各类突发事件。加大矛盾纠纷排查化解力度，健全医患纠纷、劳资纠纷、环境保护纠纷调处机制。高度重视信访工作基层基础建设，群众诉求表达渠道更加畅通。完善安全生产“一岗双责”制度，认真开展安全生产大检查和“打非治违”专项行动，市政府挂牌督办的整改项目全部完成。

　　6.民生质量继续提高。城镇居民人均可支配收入达到4.11万元，农民人均纯收入达到2.16万元，分别增长9.5%和11.2%。新增就业17.5万人,组织提供高校毕业生就业岗位14.7万个，免费培训城乡劳动者44万人，城镇登记失业率控制在2.12%，“零就业”和“零转移”贫困家庭保持动态清零。城镇职工社会保险覆盖率、城乡居民养老保险和医疗保险覆盖率均保持在99%以上，城乡老年居民社会化管理服务率超过60%，城乡居民最低生活保障指导标准由每人每月570元提高至630元。发放各类救助资金19.7亿元，惠及困难群众21万人次。建立起实行商业保险运作的社会医疗救助制度，并覆盖到城乡各类参保人员。开通居民家庭经济状况核对系统，促进社会救助制度公开公平公正。市社会福利总院建成使用。加快发展养老事业，新增养老机构床位5617张、日间照料中心75个、助餐点126个，居家养老服务体系建设全面启动。提高残疾人生活保障水平，实施“一对一”就业帮扶计划。基本建成保障性住房29893套。南环新村危旧房解危改造工程顺利交房，梅巷片区、解放新村片区危旧房改造积极推进。市区完成111万平方米老住宅小区、19万平方米零星居民楼综合整治和小区环境专项整治任务，5.3万户家庭实现天然气置换。政府确定的城区居民家庭“改厕”三年任务如期完成。城中村（无地队）改造加快实施。城镇职工住房公积金缴存覆盖面达到80.5%，职工使用住房公积金215亿元。整合公交资源，成立新的苏州市公共交通有限公司。市区新购公交车501辆，新辟公交线路14条，新增公共自行车网点702个，苏州入选国家“公交都市”创建城市。深入开展食品药品安全集中整治，张家港市成为首批国家餐饮服务食品安全示范县（市）。开展全国农产品现代流通综合试点，市区实施12个农贸市场标准化建设和改造，新增农产品配送中心8个、农产品平价商店73个、放心粮油店（柜）82个。强化价格调控和监管，居民消费价格总水平涨幅控制在2.1%。

　　在推动经济社会发展的同时，我们不断加强自身建设，努力改进政府工作。开展法治政府建设五年规划中期评估，着力解决依法行政中存在的问题。提请市人大常委会审议地方性法规议案3件，颁布政府规章5件，制定规范性文件10件，对一批政府规章和规范性文件开展了立法后评估。修订完善苏州市重大行政决策程序规定和市政府工作规则，坚持做到科学决策、民主决策、依法决策。加强行政执法规范化建设，推行行政执法事项网上办理和公开查询。抓好相对集中行政复议权试点工作，不断完善行政复议体制机制。严格执行改进工作作风、密切联系群众的各项规定，认真做好开展党的群众路线教育实践活动的思想准备和工作准备，深入实施领导干部下基层“三访三促”活动，力戒形式主义、官僚主义、享乐主义和奢靡之风，机关作风效能建设进一步加强。行政服务体系逐步完善，便民服务水平继续提高。严格控制行政支出，因公出国（境）、公务用车购置及运行、公务接待财政拨款支出下降20%。加强对资金使用和权力运行重点领域的审计，跟踪监督重大措施落实情况，财政资金使用绩效明显提高。严肃查处了一批大案要案，反腐败斗争取得新成效。市政府自觉接受市人民代表大会及其常委会的法律监督、工作监督和市政协的民主监督，坚持重大事项向市人大常委会报告、向市政协通报，注重发挥各民主党派、工商联、无党派人士和人民团体的参政议政作用。组织实施人大议案2件，办复人大代表建议245件、政协提案399件。

　　各位代表，过去一年的成绩来之不易，凝聚着全市人民的辛劳和智慧，得益于方方面面的支持和奉献。在此，我代表市政府向全市人民，向人大代表、政协委员，向离退休老同志，向各民主党派、工商联和无党派人士，向各人民团体，向驻苏解放军、武警和消防官兵，向国家和省驻苏单位，向参与、支持、关心苏州现代化建设的海内外朋友，表示衷心的感谢和崇高的敬意！

　　回顾过去的一年，我们也看到，全市经济社会发展和政府工作中仍然存在不少问题与不足，主要是：稳中求进难度加大，经济增长的质量和效益有待提高；自主创新能力仍显不足，做大新兴产业规模与化解部分行业过剩产能压力并存，转变经济发展方式任重道远；外贸形势依然严峻，部分企业生产经营困难；资源环境人口制约没有完全破解，节能减排任务相当艰巨；社会事业发展和生态文明建设存在薄弱环节，民生改善还有大量工作要做；加强社会治理、维护和谐稳定方面面临不少新情况新问题；政府职能需要加快转变，作风建设、效能建设、廉政建设必须进一步加强。今后，我们一定加倍努力，把各项工作做得更实更好更具成效。

　　二、2014年主要任务

　　根据中共苏州市委十一届六次全体（扩大）会议的部署，今年政府工作的总体要求是：全面贯彻中共十八大和十八届三中全会精神，按照中央经济工作会议和中共江苏省委十二届六次全会的部署，紧紧围绕主题主线，牢牢把握稳中求进总基调，突出深化改革创新、坚持稳增快转、提升生态质量、增进民生福祉，统筹推进扩大开放、产业升级、科技支撑、城乡一体、环境优化、文化繁荣、社会治理等重点工作，切实加强政府自身建设，努力实现经济平稳健康发展和社会全面进步。

　　全市经济社会发展的主要预期目标是：地区生产总值增长9.5%，地方公共财政预算收入增长10%以上，服务业增加值占地区生产总值的比重提高1个百分点以上，新兴产业产值占规模以上工业总产值的比重提高2个百分点左右，全社会固定资产投资和社会消费品零售总额均增长13%左右，进出口总额保持增长，实际利用外资与上年基本持平，城镇居民人均可支配收入增长10%左右，农民人均纯收入增长11%左右，居民消费价格总水平涨幅控制在3.5%左右，城镇登记失业率控制在3.5%以内，全社会研究与试验发展经费支出占地区生产总值的比重超过2.7%，单位地区生产总值能源消耗下降、主要污染物排放总量削减完成省下达任务。

　　新的一年里，我们将重点在以下八个方面下功夫、抓落实。

　　1.深化改革开放，不断增添发展动力活力。全面深化重要领域改革，努力破除体制机制弊端。围绕苏南现代化示范区建设，坚持先行先试，勇于探索创新。正确处理政府与市场的关系，使市场在资源配置中起决定性作用，更好发挥政府作用。以简政放权为核心，深化行政体制改革。继续取消和下放一批行政审批事项，凡是应该取消的都坚决予以取消，凡是可以下放给县级市、区和经济发达镇的都彻底放到位。在市级行政审批事项涉及的相关领域，探索实行负面清单管理模式，加强事中事后监管。建立各部门行政审批事项目录清单制度，并向社会公开。推进工商登记制度改革。研究制定政府购买服务指导意见和实施方案。完成卫生、计生部门机构整合和食品药品监督管理局组建工作。深入开展经济发达镇行政管理体制改革试点工作，完善机构设置和职能配置，进一步扩大经济社会管理权限。继续推动有条件的开发区和行政区实行“区政合一”管理体制。赋予苏州国家历史文化名城保护区更多的管理权限，创新和完善更加有利于古城保护与发展的体制机制。改进预算管理制度，推进政府和部门预算决算公开。优化财政支出结构，建立事权与支出责任相适应的制度。提高一般性转移支付比例，减少、合并一批专项转移支付项目。深入开展“营改增”试点。加快事业单位分类改革，推动去行政化，努力在登记管理、人事管理和建立法人治理结构等方面实现制度创新。完善国有资产管理体制，逐步建立规则统一、权责明确、分类分层的监管体系，增强监管的系统性、规范性、有效性。准确界定不同国有企业功能，完善分类考核制度，建立长效激励约束机制。推进国有企业股份制改革，鼓励国有资本、集体资本、非公有资本等交叉持股、相互融合，积极发展混合所有制经济。进一步降低准入门槛，鼓励民间资本进入法律法规未明确禁止准入的行业和领域。完善政策，加强引导，改进服务，全力营造公平竞争的市场环境，推动民营经济转型升级、健康发展。加快培育职业经理人市场，更好发挥企业家作用。以制度创新为核心，学习中国（上海）自由贸易试验区的投资管理、贸易管理、金融创新和综合监管经验，努力在有条件的区域加以借鉴和复制，争取在苏设立自由贸易试验区。争创苏州工业园区开放创新综合改革试验区，加快建设昆山深化两岸产业合作试验区。推进金融创新，优化金融生态，防范金融风险，建设区域金融中心。深化与新加坡、台湾地区的金融合作，积极争取跨境人民币业务和其他新兴金融业务的先行先试政策。鼓励和支持由民间资本发起设立中小型银行等金融机构，争取东吴企业发展银行早日获筹。充分利用互联网等新技术、新工具，创新服务模式，发展普惠金融。探索设立区域性股权交易中心，支持企业上市和发行债券。发展技术市场，完善风险投资和技术转移机制，促进科技成果资本化、产业化。开展综合性科技金融工具创新，深化保险与科技结合综合创新试点工作。

　　贯彻更加积极主动的开放战略，推动对内对外开放相互促进、“引进来”和“走出去”更好结合。实施科技兴贸战略和产业链深化工程，提高出口产品附加值和竞争力。推进加工贸易转型升级示范城市建设，力争加工贸易增值率超过85%。积极支持一般贸易，加快发展服务贸易。组织好境内外重点展会参展组展工作，帮助企业争取总部订单，扩大外贸出口。落实进口促进政策，完善张家港汽车整车进口口岸和苏州工业园区进口贸易促进创新示范区功能。办好中国（昆山）品牌产品进口交易会和中国（苏州）电子信息博览会。深入开展跨国公司总部外汇资金集中运营管理试点和国家跨境贸易电子商务服务试点工作。提高利用外资质量和效益，吸引跨国公司在苏设立区域性、功能性总部，打造外资总部集聚区。推进苏州工业园区—相城区合作经济开发区建设，创新区域联动发展模式。加大沿江港口开发建设力度，发挥综合保税区、保税港区和“港区联动”的综合功能，探索太仓港享受上海国际航运发展综合试验区相关政策的实施路径。推动企业更大步伐“走出去”，开展多种形式的对外投资，培育具有国际竞争力的跨国公司。发挥外事、侨务、对台工作的重要作用，广泛开展对外交流，办好首届世界语言大会等国际活动。抓住依托长江建设中国经济新支撑带的机遇，加强与长江中上游、中西部地区的合作。加快南北共建园区建设，做好对口援建帮扶工作。

　　2.加快转型升级，着力提升经济运行质量。以转变经济发展方式为主线，以提高经济增长质量和效益为中心，以自主创新和科技进步为动力，在保持经济平稳增长的同时，努力实现更有质量、更有效益、更可持续的发展。增加有效投入，发挥政府投资的引导作用，重点推进210项重大项目建设，年内完成投资超过2000亿元。激发民间投资活力，推动民间资本进入基础设施、基础产业、金融服务、社会服务等领域。超前谋划、储备一批战略性项目，增强投资后劲。鼓励发展社区商业、农村商业，加快传统商圈提档升级，建设新型商贸中心和特色商业街区。认真落实促进消费的各项政策措施，优化消费环境，培育消费热点，扩大信息消费，大力发展电子商务、网上交易、品牌营销、连锁经营、物流配送等商贸业态。建设国家城市旅游融合发展示范区、乡村旅游综合发展试验区和旅游度假区，推动旅游与文化、会展等相关产业融合并进。发展智慧旅游，实施旅行社行业转型升级三年行动计划，增强服务功能，扩大旅游消费。鼓励文化企业跨地区、跨行业兼并重组，促进金融资本、社会资本、文化资本相结合，提高文化产业规模化、集约化、专业化水平，带动文化消费。

　　推动产业转型，提升产业素质，打造苏州经济升级版。引导新兴产业优化发展，促进新一代信息技术、高端装备制造、新材料、新能源、生物医药、医疗器械和节能环保等产业量质齐升。帮助企业实施以新技术、新工艺、新装备、新材料推广应用为主要内容的技术改造计划，加快传统产业提档升级。推进企业信息化建设，促进信息化与工业化深度融合。以钢铁、水泥、平板玻璃等行业为重点，制定落实行动计划和年度实施方案，下大力气化解过剩产能。提高并严格执行能耗、环保和安全等行业准入标准，采取差别化资源价格政策，依法关停转改一批电镀、小化工等落后产能。推动现代服务业跨越发展，建设总投资超过600亿元的重点项目，全年服务业投资增长15%。支持服务业重点企业做大做强，做好苏州工业园区、昆山花桥国际商务城国家现代服务业综合试点工作，创建一批省级现代服务业示范区和国家级文化产业示范园区、示范基地，争创中国软件名城。坚持高标准规划、多业态发展，构建具有苏州特色的现代农业体系。进一步保护和优化“四个百万亩”，凸显多重功能、综合效益。加强现代农业园区和高标准农田建设，推动农业科技进步，新增农业园区面积6.67千公顷、“菜篮子”保供基地333公顷、省级以上农业产业化龙头企业10家。建立农业信息服务平台，健全新型农业社会化服务体系。保护和开发农业种质资源，培育地方特色优质农产品。加强粮食生产基地建设，增强粮食保供能力。

　　实施创新驱动核心战略，建设苏南自主创新先导区，发挥自主创新在转型升级中的引领和支撑作用。强化企业创新主体地位，引导创新资源向企业集聚，鼓励企业增加研发投入、建立高水平研发机构。培育中小科技企业，加快形成创新型企业集群。深化产学研合作，支持建立产业技术创新战略联盟。建设苏州国际教育园科技创业园，推动校企合作和协同创新。增强苏州自主创新广场功能，建立一批技术研发、检验检测、科技咨询服务平台，构建专业化、社会化、网络化的科技公共服务体系。推进苏州工业园区东沙湖股权投资中心、苏州高新区财富广场等创业投资集聚区建设，扩大“科贷通”资金规模和覆盖范围。加强知识产权运用和保护，建设国家知识产权示范城市。认真开展技术标准化工作，争创全国质量强市示范城市。实施各项人才计划，重点引进国家“千人计划”、省“双创计划”人才和高水平创新创业团队，建好中国苏州人力资源服务产业园，继续办好国际精英创业周和“赢在苏州”国际精英创新创业大赛，完善人才柔性流动机制，深化产教融合，注重培养高技能人才，全年新增各类人才16万人。

　　3.强化统筹协调，深入推进城乡一体发展。坚持工业企业向镇级及以上工业园区集中为主、农民居住向城镇集中为主、承包地流转以转给集体经济组织为主、经营主体以本地农民为主，努力提升城乡规划、现代农业、节约集约、强村富民、美丽镇村、工农互惠水平。推进家庭经营、集体经营、合作经营、企业经营等共同发展，加快构建立体式、复合型现代农业经营体系，注重培养新型职业农民。鼓励和引导工商资本到农村发展适合企业化经营的现代种养业，向农业输入现代生产要素和经营模式。严格落实耕地保护各项制度，坚决遏制违法用地行为。争取建立城乡建设用地空间置换制度，进一步用好城乡建设用地增减挂钩试点政策。以优化建设用地空间布局保障发展，以优化农用地结构布局保护耕地，以优化镇村居住用地布局保护权益。巩固农村集体土地确权登记成果，探索农村宅基地腾退流转机制。积极稳妥扎实推进以人为核心的城镇化，重点解决已经转移到城镇就业的农业转移人口落户问题，加快实现农业转移人口市民化。建设农民集中居住安置房，农民集中居住率提高到56%以上。支持集体经济组织联合发展、异地发展、转型发展，积极参与工业化、信息化、城镇化和农业现代化建设，农村集体总资产增长12%，村级集体经济收入增长10%以上。加强集体资产、资金、资源管理，建立农村集体资产信息监管平台，探索开展农村集体资产产权交易。维护农民生产要素权益，赋予农民更多财产权利。深化股份合作经济股权固化改革，鼓励农民增资扩股，提高农民财产投资性收入比重，巩固农民长效增收机制。做大城乡一体化建设基金，发展农村金融组织，完善农业保险体系，扩大农业担保规模。统筹小城镇和新农村建设，进一步优化镇村布局，推动公共财力向农村倾斜、公共服务向农村延伸、公共管理向农村覆盖。完成保留村庄规划编制工作，有计划地进行村庄改造。持续抓好“美丽镇村”建设，促进古镇、古村落保护和发展，充分彰显江南水乡风貌。

　　4.坚持建管并举，全面优化城市功能品质。着手编制新一轮苏州市城市总体规划，科学制定各类分区规划、详细规划、专项规划和城市设计。按照中心城市“一核四城”的定位，优化空间布局、功能布局和产业布局，更好发挥苏州国家历史文化名城保护区功能，提升东部综合商务城发展水平，推进西部生态科技城、南部太湖新城和北部高铁新城建设。启动建设沪通铁路苏州段。续建常嘉高速公路昆山至吴江段、张家港疏港高速公路和312国道苏州段分流线。基本建成中环快速路及市区内环快速路延伸线，新建和改造一批城市道路。加快建设轨道交通4号线及支线、2号线延伸线和苏州高新区有轨电车1号线，开工建设轨道交通3号线。增强苏州港集散功能，扩大货物吞吐量。基本完成苏南运河苏州段航道整治工程，加大杨林塘、申张线航道整治力度。推进虎丘地区综合改造和桃花坞历史文化片区、天赐庄片区、渔家村越城遗址保护整治利用工程，修缮保护古建老宅。推动1000千伏特高压交流工程建设，加快为苏州接入大功率清洁能源。认真开展全国电力需求侧管理城市综合试点工作。深入实施信息化发展规划，落实“宽带中国”苏州实施方案，推进“智慧苏州”重点项目，加快信息基础设施建设。扩大4G网络覆盖面，提升应用服务水平。科学制定设防标准，调整优化圩区布局，增强城市防洪能力。加强自然灾害监测预警，增设应急避难场所。抓好人防工程建设，新增人防设施90万平方米。建成数字城市地理空间框架，开展全市地理国情普查。综合整治城郊结合部、城中村、棚户区、老旧小区、背街小巷、城市河道、低洼易淹易涝片区、建设工地、农贸市场，改造美化市区人民路沿线街景容貌，规范管理占道经营、车辆停放、户外广告，提升城市长效管理水平，打造最干净、最整洁城市。

　　5.建设生态文明，大力营造优美宜居环境。坚持绿色发展、循环发展、低碳发展，努力形成与生态文明相适应的发展方式，争创国家生态文明建设试点示范市和国家生态园林城市。扎实推进生态文明建设“十大工程”，进一步优化建设内容。加快实施太湖流域水环境综合整治、阳澄湖生态优化和七浦塘综合整治、古城河道水质提升等工程，落实长效管理措施。严格保护水源地，确保饮用水安全。建设和改造城乡污水处理系统，全面提高生活污水处理率。实施“蓝天工程”，加强煤炭消耗总量控制，全面整治各类燃煤锅炉，严格落实重点行业特别排放限值，加速淘汰老旧车船，着力提高城乡保洁和建筑施工防尘水平，继续强化秸秆禁烧和综合利用措施，推进区域性、复合型、项目化大气污染治理。严肃查处噪声污染，营造安静舒适的人居环境。综合防治重金属污染，确保工业危险废物全部得到安全处置。加强能源消耗源头控制，组织实施节能重点工程和节能技术推广应用示范项目，开展能效对标行动，降低单位产出能源消耗。深化国家低碳城市试点工作，推动新能源汽车、燃气发电和低碳技术应用，探索建立碳排放交易平台，支持开发区争创低碳工业园区。实施开发区整体循环化改造，积极发展循环经济。扩大生活垃圾分类试点范围，建成建筑垃圾资源化利用终端处置项目，提高餐厨垃圾和绿化垃圾规范处置率，做好动物无害化处理工作。完善能源、水、土地节约集约使用制度，提高资源利用效率。推进“两河一江”、“两山一镇”环境综合整治，建设上方山石湖生态园、吴中区临湖园博园，筹备江苏省第九届园艺博览会。持续开展“绿色苏州”建设，提高绿化质量，更好发挥生态功能。兴建一批郊野公园，为苏州永久性地留下绿色空间。市区新增绿地500万平方米，农村新增林地绿地3.33千公顷，全市陆地森林覆盖率达到29.35%。全面加强湿地保护，继续建设虎丘湿地公园、太湖湿地公园，做好重要湿地勘界执法和湿地公园创建升级工作。开展土壤污染调查与修复，提高山体整治复绿水平。加强重要生态功能区和生物多样性保护，增强生态产品生产能力。坚定不移实施主体功能区制度，完善生态环境保护管理和责任追究制度，坚守生态保护红线。贯彻落实生态补偿和政策激励制度，强化谁保护谁受益、谁损害谁补偿的利益导向。及时公布环境信息，健全举报制度，加强社会监督。

　　6.繁荣各项事业，切实增强社会治理能力。全面发展社会事业，更好满足人民需求。深化教育领域综合改革，建设教育现代化示范区。做好新一轮教育规划布局调整工作，推进城乡教育一体化发展。加快建设和改造中小学、幼儿园，实施新三年校舍安全工程和外来工子弟学校提档升级工程。注重基础教育、职业教育、高等教育和继续教育之间的有效衔接，构建人才培养“立交桥”。完善政府补贴、政府购买服务等制度，鼓励社会力量兴办教育。加强国际交流合作，广泛利用优质教育资源。坚持立德树人，深化素质教育。加大校长和教师培训、交流力度，构建教育人才高地。推动全民学习、终身学习，建设学习型社会。完善文化管理体制和公共文化服务体系，扩大公共文化产品供给。建成中国昆曲剧院和苏州档案馆新馆，推进苏州图书馆二期工程、现代传媒广场、苏州新闻大厦和苏州文博中心、苏州高新区文体中心建设，改造苏州丝绸博物馆，筹建中国丝绸档案馆。实施舞台艺术精品工程，开展系列文化惠民活动。注重文化传承，延续历史文脉。完善非物质文化遗产保护四级名录，向联合国教科文组织申报“创意城市网络—手工艺和民间艺术之都”。出版发行《苏州市志》。倡导全民阅读，创建全国“书香城市”。全面推进县级公立医院综合改革，巩固完善基本药物制度，落实各类公共医疗卫生服务项目。建成苏州大学附属第一医院主体迁建一期工程、苏州大学附属儿童医院园区总院，建设市公共医疗中心和独墅湖医院、苏州科技城医院、姑苏医院，筹建苏州市第九人民医院。实施婴幼儿健康促进工程，做好医疗自助服务进家庭工作。健全城乡社区卫生服务运行机制，逐步推行分级诊疗。强化疾病防控，提高重大疫情和公共卫生突发事件应急处置能力。启动实施一方是独生子女的夫妇可生育两个孩子的政策，开展生育关怀、服务到家惠民活动。建成市民健身中心体育场、体育馆，建设苏州工业园区体育中心，积极筹备第53届世界乒乓球锦标赛。落实妇女儿童“十二五”发展规划，建设市妇女儿童活动中心迁建工程。推进民族团结进步事业发展，提高依法管理宗教事务水平。拓展军队保障社会化领域，建设国防动员训练基地，推动军民融合深度发展。

　　提高社会文明程度，促进社会和谐有序。深入开展城乡文明一体化创建工作，争创全国首个文明城市群。改进社会治理方式，深化“政社互动”，激发社会组织活力，鼓励和支持志愿服务。建设法治城市，争创法治建设先导区。加强法制宣传教育，增强法制观念，提高法治意识。完善法律顾问制度、法律援助制度和社区矫正制度。健全矛盾纠纷调处化解机制，妥善解决人民群众关心的公共安全、权益保障、公平正义等问题。畅通信访渠道，及时就地回应群众合理诉求，提高信访事项办理效能。加强诚信体系建设，建成“诚信苏州”网和政府、企业、个人信用信息基础数据库。实施“平安苏州”建设信息化提升工程，完善立体化、现代化社会治安防控体系，依法严密防范和惩治违法犯罪活动，高度警惕和坚决防范敌对势力的分裂渗透颠覆活动，打造“平安中国”建设示范区。加强互联网管理，维护网络和信息安全。保持安全生产监管高压态势，健全隐患排查治理和安全预防控制体系，落实企业安全生产主体责任，坚决遏制重特大安全事故。建立应急物资储备库、应急救援专家库和公共应急信息发布系统，确保及时有效处理各类突发事件。

　　7.突出民生优先，持续提高人民生活水平。把促进社会公平正义、增进民生福祉作为政府工作的出发点和落脚点，全力办好民生实事，让发展成果更多更公平惠及全市人民。认真实施城乡居民收入倍增计划，落实城乡低收入家庭增收措施。健全工资决定和正常增长机制，完善资本、知识、技术、管理等由要素市场决定的报酬机制。做好“零就业”、“零转移”贫困家庭就业援助工作，开发公益性岗位8000个。高度重视高校毕业生就业工作，确保苏州籍高校毕业生就业率超过95%。继续开展城乡劳动者免费培训。完善创业扶持政策，深化国家创业型城市建设。创新劳动关系协调机制，畅通职工表达合理诉求渠道。稳步提高社会保障水平，确保城镇职工社会保险覆盖率、城乡居民养老保险和医疗保险覆盖率巩固在99%以上，城乡老年居民社会化管理服务实现全覆盖。发展企业年金、职业年金、商业保险，构建多层次社会保障体系。加大对“支出型”贫困家庭的救助力度，扩大低保边缘重病困难对象救助范围。支持慈善事业发挥扶贫济困积极作用，推动红十字事业健康发展。加强养老服务设施建设，新增养老机构床位3800张，建成市老年活动中心和市怡养老年公寓，发展居家养老和老年服务产业。健全残疾人权益保障制度，提高社会化服务水平。大力推进梅巷片区、解放新村片区危旧房改造。建设保障性住房，年内新开工29491套。做好住房公积金扩面工作，力争城镇职工缴存覆盖面超过85%。落实公交优先发展新三年行动计划，推进全球环境基金缓解大城市拥堵、减少碳排放项目，市区新购新能源和清洁能源公交车600辆，新增出租车500辆。采取一系列政策措施，下大力气缓解交通拥堵问题。深入开展放心消费创建活动，引导健康文明消费。更加注重农产品质量和食品药品安全，理顺体制、完善机制、加强监管，抓好集中治理整顿。加快现代农产品物流园建设和农贸市场标准化改造，新增一批农产品配送中心、直供网点和放心粮油店（柜）。健全粮食批发市场体系，全面完成地方粮食储备任务。完善价格调控机制，加强市场价格监管，保持物价总体稳定。

　　8.加强自身建设，努力打造人民满意政府。加快转变政府职能，创新行政管理方式，增强政府公信力和执行力，建设法治政府和服务型政府。按照“照镜子、正衣冠、洗洗澡、治治病”的总要求，认真开展以为民务实清廉为主要内容的党的群众路线教育实践活动，抓好学习教育、听取意见，查摆问题、开展批评，整改落实、建章立制等重点环节，确保在党员干部深受教育、突出问题得到解决、作风形象明显改进、人民群众真正满意等方面取得实效。不折不扣贯彻改进工作作风、密切联系群众的各项规定，持之以恒反对形式主义、官僚主义、享乐主义和奢靡之风。坚持依法行政，严格按照法定权限和法定程序行使权力、履行职责。高质量起草地方性法规草案，制定政府规章和规范性文件，组织开展立法后评估，做好行政复议工作。完善行政执法程序，规范执法自由裁量权，加强对行政执法的监督，做到严格规范公正文明执法。建立健全科学有效的权力制约和协调机制，构建决策科学、执行坚决、监督有力的权力运行体系。推行政府及工作部门权力清单制度，完善政务公开制度，推进决策公开、管理公开、服务公开、结果公开。健全政务服务体系，进一步落实领导干部下基层“三访三促”等制度，扎实做好服务群众、服务企业、服务基层的各项工作。坚决执行《党政机关厉行节约反对浪费条例》，大力压减各类会议，从严控制会议规格，精简文件简报资料，切实改进文风会风，严控创建达标检查评比和节庆论坛等活动，继续压缩“三公”经费支出，建设节约型机关。加强公务员管理，抓好思想道德教育和业务能力培训，全面提升服务意识和作风效能。严格落实党风廉政建设责任制，健全惩治和预防腐败体系，始终保持惩治腐败的高压态势，努力实现干部清正、政府清廉、政治清明。强化公共资金、国有资产、国有资源审计，更加注重对民生资金、实事项目和“三公”经费的跟踪监督，深化经济责任审计，加大审计结果公开力度。依法规范招投标活动。加强地方政府性债务管理，严格举债程序，防控债务风险。高质量完成第三次经济普查工作。各级政府要自觉接受人民代表大会及其常委会的法律监督、工作监督，接受人民政协的民主监督，接受司法机关的监督，接受新闻舆论和社会公众的监督，认真实施人大议案，办理代表建议和政协提案。

　　各位代表，苏州改革发展任务十分繁重，做好新一年工作责任重大。让我们紧密团结在以习近平同志为总书记的党中央周围，深入贯彻落实中共十八大和十八届三中全会精神，在中共苏州市委的坚强领导下，抢抓机遇、深化改革，齐心协力、埋头苦干，为夺取“三区三城”建设新胜利、率先基本实现现代化而努力奋斗！
